# Infrahepatic Inferior Vena Cava Clamping does not Increase the Risk of Pulmonary Embolism Following Hepatic Resection

**DOI:** 10.1007/s00268-021-06159-4

**Published:** 2021-05-28

**Authors:** Emrullah Birgin, Arianeb Mehrabi, Dorothée Sturm, Christoph Reißfelder, Jürgen Weitz, Nuh N. Rahbari

**Affiliations:** 1 Department of General, Visceral and Transplantation Surgery, University of Heidelberg, Heidelberg, Germany; 2 Department of Gastrointestinal, Thoracic and Vascular Surgery, University Hospital Carl Gustav Carus at the Technische Universität Dresden, Dresden, Germany; 3 Present Address: Department of Surgery, Medical Faculty Mannheim, University Medical Center Mannheim, Heidelberg University, Theodor-Kutzer-Ufer 1-3, 68167 Mannheim, Germany

## Abstract

**Background:**

Infrahepatic inferior vena cava (IVC) clamping reduces central venous pressure. However, controversies remain regarding its impact on postoperative complications, particularly, the incidence of postoperative pulmonary embolism (PE). The aim of the study was to determine the impact of IVC clamping on the incidence of PE in patients undergoing hepatectomy.

**Methods:**

A pooled analysis of five prospective trials on patients who underwent hepatic resection over a period of 10 years was performed. Patients with infrahepatic IVC clamping were compared to patients without infrahepatic IVC clamping. Outcomes were studied by univariate and multivariate analyses.

**Results:**

Of 505 included patients, 141 patients had IVC clamping and 364 patients served as control group. The rate of postoperative PE was comparable between groups (3% vs. 3%; *P* = 0.762), as were postoperative morbidity (*P* = 0.932), bile leakage (*P* = 0.272), posthepatectomy hemorrhage (*P* = 0.095), and posthepatectomy liver failure (*P* = 0.605), respectively. No clinicopathological and intraoperative risk factors were found to predict the onset of PE. Subgroup analyses of patients with major hepatectomy and vascular resections confirmed no adverse perioperative outcomes to be associated with IVC clamping.

**Conclusions:**

Infrahepatic IVC clamping does not increase the incidence of postoperative PE.

## Introduction

Hepatic resection is the treatment of choice for benign and malignant liver tumors [1, 2]. Despite substantial improvements in perioperative mortality within the past three decades, morbidity after hepatic resection remains high with rates up to 60% [3]. There is a strong association of postoperative complications with the amount of intraoperative blood loss [4]. Thus, various intraoperative strategies to limit blood loss were developed including low central venous pressure (CVP) during hepatic transection and vascular occlusion techniques [5, 6]. Infrahepatic clamping of the inferior vena cava (IVC) is a method of outflow control to maintain a low CVP. Compared to the commonly applied anesthesiological approach to lower CVP by fluid restriction, IVC clamping can be used in euvolemic patients with the advantage of less hemodynamic instability and improved organ perfusion [7]. In a previous randomized controlled trial, we found IVC clamping to be effective in lowering CVP and reducing intraoperative blood loss. However, it was also associated with a significantly higher incidence of postoperative pulmonary embolism (PE) [8]. Indeed, several other randomized controlled trials did not show any morbidity related to the use of infrahepatic IVC clamping during hepatectomy, though heterogeneous surgical techniques were applied with respect to routine use of inflow control and the extent of IVC clamping [9–12]. Furthermore, two recent meta-analyses of randomized controlled trials, mainly involving cohorts with primary liver malignancies, suggested no adverse events following IVC clamping within the above-mentioned limitations [13, 14].

Due to the heterogeneous data, it was the aim of the present analysis to assess the impact of IVC clamping on postoperative complications and in particular the incidence of PE in patients undergoing hepatic resection. To obtain highly valid and reliable data, we used individual patient data from registered and published trials that documented the use and duration of infrahepatic IVC clamping during elective liver resection in a prospective fashion.

## Materials and methods

This study was performed as a secondary data analysis of four randomized controlled trials (NCT00732979, NCT01049607, NCT01858987, NCT02612220) and one prospective cohort study (NCT01073345) conducted between April 2007 and September 2017 at the Department of General, Visceral and Transplantation Surgery, University of Heidelberg, and the Department of Gastrointestinal, Thoracic and Vascular Surgery, University Hospital Carl Gustav Carus, Technische Universität Dresden [8, 15–18]. These trials were selected due to the use of consistent surgical techniques in these institutions with or without the use of continuous (non-intermittent) IVC clamping during hepatectomy in primary and secondary liver malignancies. The included trials applied inflow control (Pringle maneuver) only in case of severe bleeding (and documented as secondary endpoint) while the perioperative care was identical in each case. Five other published trials on the use of IVC clamping were excluded due to (1) the application of IVC clamping in highly selective patient cohorts (e.g., with large hepatocellular carcinoma) [19], the restricted use of modified surgical hepatectomy techniques (e.g., anterior-approach) [11], (2) the concomitant use of Pringle maneuver with IVC clamping in all liver resections [9, 10], and (3) the use of a partial IVC clamping technique instead of a continuous IVC clamping technique [12]. The study was done in accordance with the Principles of Good Practice of Secondary Data Analysis (GPS). The present cohort study with secondary data analysis using de-identified data did not require an Institutional Review Board review according to our local institutional review policy.

### Patient eligibility criteria and data extraction

Patient eligibility criteria for the individual trials were reported in the original publications. Patients were included in the present analysis in case hepatic resection was carried out and data on the use of infrahepatic IVC clamping were available. Patients who required infrahepatic IVC clamping for the purpose of IVC resection were excluded. The following data were extracted from the individual databases for the purpose of the present analysis: age, gender, body mass index (BMI), American Society of Anesthesiologists (ASA) score, diagnosis, presence of liver steatosis, presence of liver fibrosis, presence of liver cirrhosis, history of chemotherapy, history of hepatic resection, perioperative laboratory tests including bilirubin, aspartate aminotransferase, alanine aminotransferase, alkaline phosphatase (AP), gamma glutamyltransferase, creatinine, hemoglobin, platelets, and international normalized ratio (INR). In addition, the following operative details were extracted: extent and type of resection, number of resected segments, creation of bilioenteric anastomosis, and technique of hepatic parenchymal transection.

### Definitions and outcomes

The primary endpoint was the incidence of postoperative PE. Postoperative PE was defined as pulmonary arterial obstruction and confirmed by spiral computed tomography of the chest in all suspected cases presenting with respiratory insufficiency [20]. Routine and scheduled computed tomography scans to rule out PE were not performed in the included studies [7, 8]. All patients with PE were transmitted to the intermediate/or intensive care unit and treated with therapeutic doses of low molecular weight or unfractionated heparin. Thromboprophylaxis was performed in the studies in line with the German guidelines on prophylaxis of thromboembolism which was initially published in 2003 [21]. In brief, all patients used elastic stockings and thromboprophylaxis with low molecular weight or unfractionated heparin irrespective of intraoperative vascular resections or type of surgery. The Clavien-Dindo classification was used to document the severity of postoperative complications. Clinically relevant complications were defined as Clavien-Dindo complications grade III and higher. Posthepatectomy bile leakage, posthepatectomy liver failure, and posthepatectomy hemorrhage were recorded in line with the definitions by the International Study Group of Liver Surgery (ISGLS) [22]. Postoperative medical complications included the frequency of PE, cardiac complications, deep vein thrombosis, and acute renal failure. In addition, the following variables and outcomes were considered in the present analysis: need for and duration of portal triad clamping, operating time, total blood loss, perioperative transfusion, postoperative hospital stay, interventional drainage, reoperation, and mortality within 90 day of surgery.

### Study interventions and perioperative care

Hepatic resections were performed in a standardized fashion as outlined in the study publications [8, 15–18]. In brief, resections were carried out via a laparotomy under low CVP (< 5 mmHg) without routine use of vascular inflow control (only in case of significant intraoperative blood loss). Parenchymal transection was achieved by clamp-crushing technique, stapler, ultrasonic dissection, or a sealing energy device. Topical agents and argon beam coagulation were used at the discretion of the surgeon.

Except for the randomized trial on infrahepatic IVC clamping, clamping of the IVC was performed at discretion of the surgeon and anesthesiologist. It was carried out below the hepatoduodenal ligament and above the right renal vein using a vascular clamp. Initially, the infrahepatic IVC was clamped for a short period. In case the patient tolerated occlusion of the IVC, the vascular clamp was applied for complete clamping of the infrahepatic IVC for the entire period of hepatic parenchymal transection. Infrahepatic IVC clamping was applied on the study group only.

### Statistical analysis

Categorical variables were summarized by absolute and relative frequencies (percentage) and compared using Pearson’s *χ*^2^ or Fisher’s exact test. Continuous variables were expressed as mean (standard deviation) or median (interquartile range) and compared with Student’s *t*-test or *Wilcoxon-Mann–Whitney* test depending on the pattern of distribution. The Holm-Sidak method was used to adjust for multiple *t*-testing. A bivariate logistic regression analysis of variables (*P* < 0.05) was performed to determine risk factors of postoperative PE. A generic inverse-variance method was conducted using a fixed-effects and random effects model to assess differences between the individual studies for the rate of PE. Odds ratios (ORs) with 95% CI were calculated for binary outcomes. The interstudy heterogeneity (*I*^*2*^) was assessed using the *I*^*2*^ value. Subgroup analyses were performed for patients with major hepatectomy with and without vascular resections. A two-sided *P* < 0.05 was deemed statistically significant. Statistical analysis was performed using R version 3.6.1.

## Results

### Patient characteristics

**Fig. 1:**
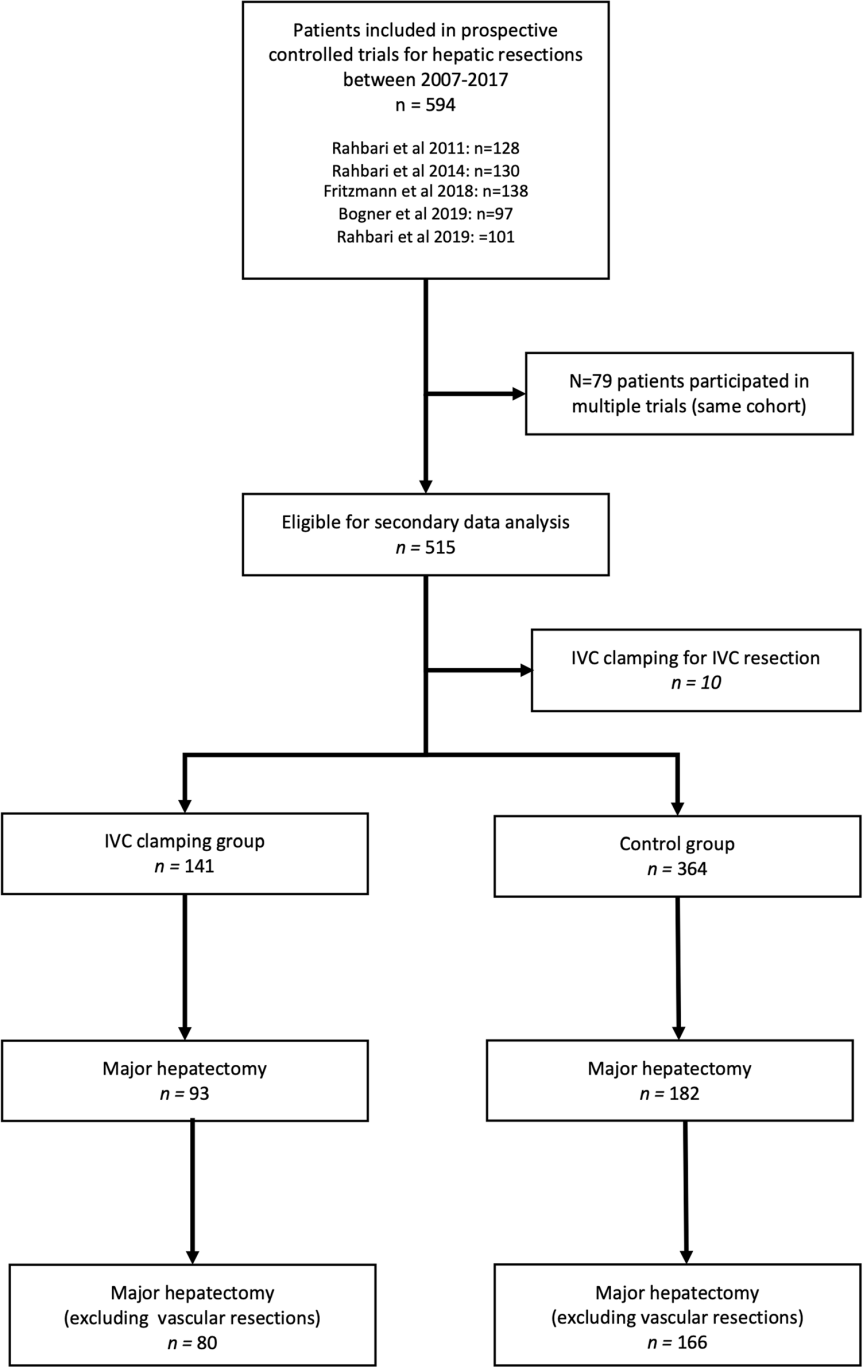
Study flow diagram

Of 594 patients who were included in prospective controlled trials, a total of 505 patients met the inclusion criteria. Of these, 141 patients received IVC clamping and 364 patients served as control group. The study flow diagram is shown in Fig. 1. Patients’ baseline characteristics are outlined in Table 1. Patients in the IVC clamping group were younger (59 $\pm$ 12 vs. 62 $\pm$ 12, *P* = 0.011) compared to the control group. Other baseline characteristics were similar in both groups.

**Table 1:** Baseline characteristics

	Infrahepatic IVC clamping (*n* = 141)	Control (*n* = 364)	*P* value
Age (years)*	59 (12)	62 (12)	0.011
BMI (kg/m^2^)*	26 (4)	26 (5)	0.189
Sex ratio (male:female)	80:61	222:142	0.382
ASA			0.566
I	4 (3)	7 (2)	
II	63 (45)	142 (38)	
III	74 (52)	212 (58)	
IV	0	1 (1)	
Missing	–	2 (1)	
Steatosis			0.169
No	50 (35)	98 (27)	
Grade 1 + 2	84 (60)	249 (68)	
Grade 3 + 4	5 (4)	15 (4)	
Missing	2 (1)	2 (1)	
Fibrosis			0.576
No	67 (48)	186 (51)	
Grade 1 + 2	65 (46)	155 (42)	
Grade 3	8 (5)	22 (6)	
Missing	1 (1)	1 (1)	
Liver cirrhosis			0.549
No	128 (91)	314 (86)	
Child A	12 (8)	45 (12)	
Child B	1 (1)	5 (2)	
History of chemotherapy	61 (43)	176 (49)	0.321
History of hepatic resection	22 (16)	84 (23)	0.068
Diagnosis			0.116
Primary liver malignancy	47 (33)	114 (31)	
Secondary liver malignancy	75 (53)	221 (61)	
Benign liver disease	19 (14)	29 (8)	
Preoperative laboratory tests*			
Bilirubin (mg/dl)	0.8 (1.4)	0.8 (1.8)	0.828
AP (U/l)	164 (161)	147 (154)	0.329
gGT (U/l)	195 (300)	169 (271)	0.372
AST (U/l)	38 (39)	39 (34)	0.801
ALT (U/l)	44 (51)	42 (46)	0.627
Creatinine (mg/dl)	0.8 (0.2)	0.8 (0.3)	0.293
Hemoglobin (g/dl)	13 (2)	13 (2)	0.205
Platelets (/nl)	275 (120)	275 (107)	0.922
International normalized ratio	1.0 (0.1)	1.0 (0.1)	0.232

Values in parentheses are percentages unless indicated otherwise;*Values are mean (s.d.)

*IVC* inferior vena cava, *ASA* American Society of Anesthesiologists, *BMI* body mass index, *AP* alkaline phosphatase, *gGT* gamma glutamyl transferase, *AST* aspartate aminotransferase, *ALT* alanine aminotransferase

### Operative details and intraoperative outcome

The mean duration of IVC clamping in the clamping group was 17 $\pm$ 16 min with a total of 22 patients (16%) having a clamping-time below 5 min. Characteristics of surgery and intraoperative outcomes are summarized for both study groups in Table 2. CVP prior to resection was similar in both groups (5 $\pm$ 3 vs. 5 $\pm$ 4; *P* = 0.848). There were significantly more major liver resections (66% vs. 50%, *P* = 0.001) and a trend for more vascular resections (9% vs. 6%, *P* = 0.096) in the IVC clamping group. As expected, the Pringle maneuver was applied more frequently in the IVC clamping group due to severe intraoperative bleeding, though this did not reach statistical significance (23% vs. 19%, *P* = 0.113). Stapler and ultrasound-based devices were more frequently applied in the IVC clamping group compared to the control group, whereas more sealing devices were used for parenchymal dissection in the control group (*P* < 0.001). Despite a higher percentage of major hepatectomies and vascular resections in the IVC clamping group, there were no significant differences in intraoperative blood loss, operating time and the need for intraoperative blood transfusion between both groups.

**Table 2:** Operative details and intraoperative outcome

	Infrahepatic IVC clamping (*n* = 141)	Control (*n* = 364)	*P* value
CVP prior resection *	5 (3)	5 (4)	0.848
Extent of resection			0.001
Major hepatectomy	93 (66)	181 (50)	
Minor hepatectomy	48 (34)	183 (40)	
Type of resection			0.010
Right/extended right hemihepatectomy	52 (37)	117 (32)	
Left/extended left hemihepatectomy	37 (26)	56 (15)	
Central hepatectomy	2 (1)	8 (2)	
Anatomic resection > 2 segments	4 (4)	2 (1)	
Anatomic resection ≤ 2 segments	29 (20)	87 (24)	
Non-anatomical resections	17 (12)	94 (26)	
No. of resected segments †	4 (2–5)	4 (1–4)	0.001
Extrahepatic resection	17 (13)	47 (13)	1.000
Vascular resection	13 (9)	18 (5)	0.096
Portal vein	9 (6)	8 (2)	
Hepatic artery	3 (2)	2 (1)	
Portal vein + hepatic artery	1 (1)	2 (1)	
Hepatic vein	0	6 (1)	
Bilioenteric anastomosis	24 (16)	50 (14)	0.400
Resection device			< 0.001
Crush clamp	21 (15)	56 (15)	
Stapler	78 (55)	159 (43)	
Ultrasound-based	15 (11)	13 (4)	
Sealing device	27 (19)	134 (37)	
Missing	–	2 (1)	
Pringle maneuver	36 (23)	69 (19)	0.113
Duration of pringle maneuver (min)*	12 (9)	16 (15)	0.145
Operating time (min)*	205 (100)	210 (102)	0.685
Total blood loss (ml)†	800 (450–1400)	800 (500–1400)	0.679
Mean blood loss*	1100 (1000)	1100 (1000)	0.956
Intraoperative transfusion	25 (18)	59 (16)	0.274
PRBCs†	2 (2–4)	2 (2–4)	0.500
FFP†	4 (3–5)	4 (2–4)	0.103
MABP prior resection*	77 (13)	76 (13)	0.323
SBP prior resection*	108 (19)	108 (20)	0.600

Values in parentheses are percentages unless indicated otherwise; ^†^Values are median (iqr); *Values are mean (s.d.)

*IVC* inferior vena cava, *CVP* central venous, *PRBC* packed red blood cell, *FFP* fresh frozen plasma, pressure. *MABP* mean arterial blood pressure, *SBP* systolic blood pressure

### Incidence of postoperative PE

**Fig. 2:**
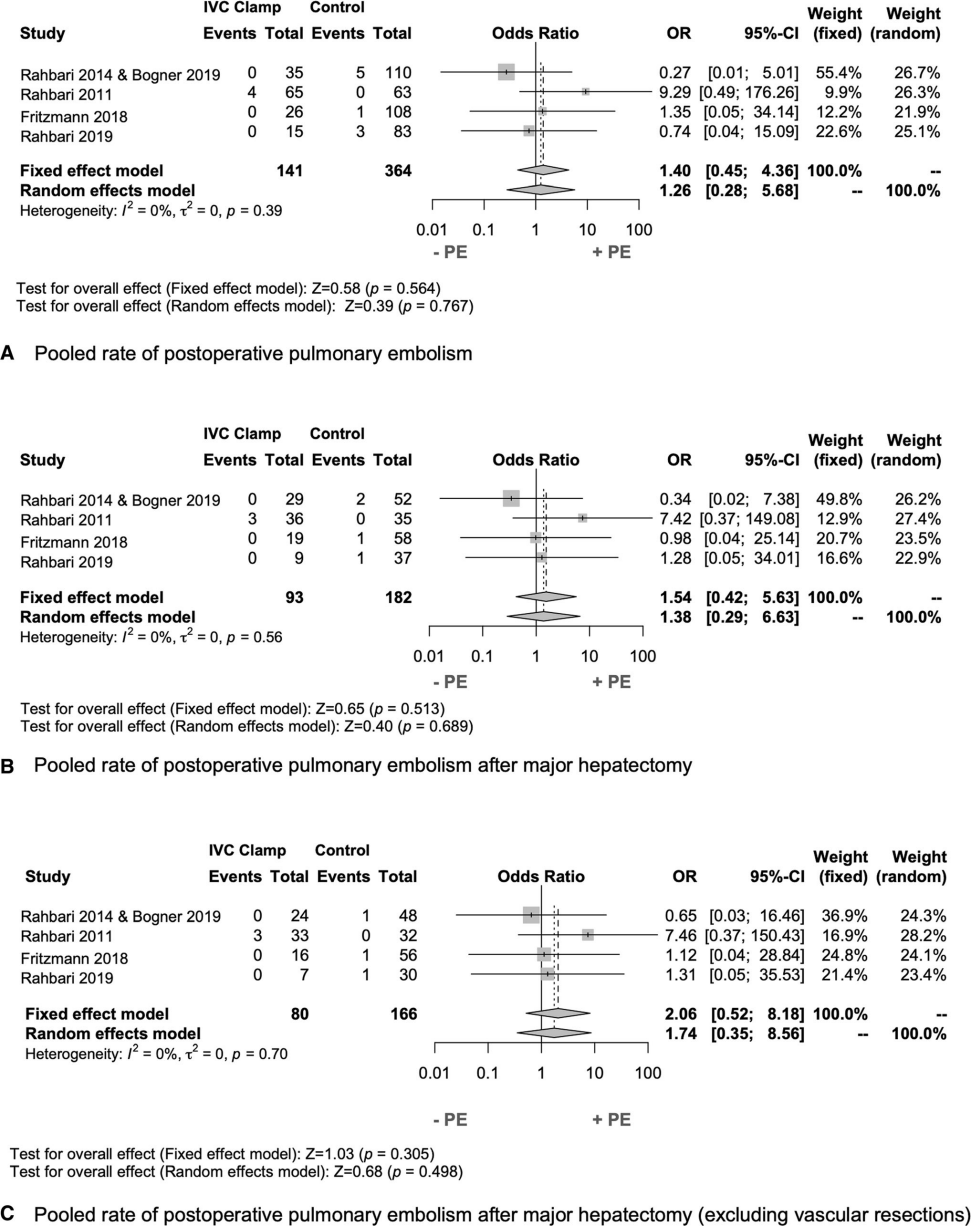
Forrest plot of the rate of postoperative pulmonary embolism The pooled rate of postoperative pulmonary embolism (PE) in the infrahepatic IVC clamping and control group is shown in the total cohort (**a**), in the major hepatectomy cohort (**b**), and in the major hepatectomy cohort without vascular resections (**c**). An inverse-variance random effects model was used for pooling of the rate of PE in the studies. Squares and horizontal bars indicate point estimate (odds ratios) with 95% CI for the individual studies

Analysis of the primary endpoint revealed no significant difference in the rate of postoperative PE between the study groups (3% vs. 3%, *P* = 0.762). The pooled rate of PE in the individual studies using fixed and random effects models indicated the incidence of PE after infrahepatic IVC clamping to be limited to a single study only (*P* = 0.564 and *P* = 0.767) (Fig. 2A). Next, further risk factors for the onset of PE were determined by univariate analysis. ASA $\ge$3 (*P* = 0.043), INR $<$ 0.9 (*P* = 0.016), and intraoperative transfusion of fresh frozen plasma (FFP) (*P* = 0.017) were associated with postoperative PE. The total number of intraoperative transfused FFPs were comparable in patients with PE and without PE (4 (3–4) vs. 4 (2–4), *P* = 0.879). On multivariate analysis, none of these factors were independently associated with PE (Table 3).

**Table 3:** Clinicopathological factors associated with postoperative pulmonary embolism

	Univariate			Multivariate	
	PE (*n* = 13)	No PE (*n* = 492)	*P* value	OR (95%CI)	*P* value
Age (years)*	65 (11)	61 (12)	0.166		
BMI (kg/m^2^)*	27 (3)	26 (5)	0.419		
Sex ratio (male:female)	7:6	295:197	0.657		
ASA $\ge$ III	11 (85)	278 (57)	0.043	3.09 (0.64–14.99)	0.162
Steatosis grade $\ge$ 3	0	24 (5)	0.412		
Fibrosis grade $\ge$ 3	1 (8)	29 (6)	0.790		
Liver cirrhosis	2 (15)	61 (12)	0.748		
Primary or secondary liver malignancies	10 (77)	447 (91)	0.091		
History of hepatic resections	2 (15)	104 (21)	0.615		
History of chemotherapy	6 (46)	231 (47)	0.955		
Platelets $\ge$ 450/nl	0	36 (8)	0.301		
INR < 0.9	1 (8)	4 (1)	0.016	NA	
Preoperative laboratory tests					
Bilirubin (mg/dl)	0.6 (0.4)	0.8 (1.6)	0.597		
Creatinine (mg/dl)	0.8 (0.1)	0.9 (0.3)	0.326		
AP (U/l)	147 (135)	152 (156)	0.914		
gGT (U/l)	204 (221)	175 (281)	0.713		
AST (U/l)	38 (34)	39 (36)	0.926		
ALT (U/l)	38 (25)	43 (49)	0.690		
Hemoglobin (g/dl)	13 (2)	13 (2)	0.825		
CVP prior resection *	6 (4)	5 (4)	0.611		
Major hepatectomy	7 (54)	267 (54)	0.976		
No. of resected segments †	4 (4–5)	4 (4–5)	0.675		
Vascular resection	1 (8)	30 (6)	0.813		
Extrahepatic resection	2 (15)	62 (13)	0.784		
Bilioenteric anastomosis	4 (31)	70 (14)	0.096		
IVC clamping	4 (31)	137 (28)	0.817	1.55 (0.42–5.70)	0.506
Intraoperative transfusions					
PRBC	2 (15)	82 (17)	0.902		
FFP	3 (30)	33 (8)	0.017	3.99 (0.96–16.23)	0.055
Pringle maneuver	0	105 (21)	0.061		
Operating time (min)*	226 (70)	206 (100)	0.481		
Total blood loss (ml)†	1000 (700–1400)	800 (400–1400)	0.203		
Mean blood loss (ml)*	1200 (700)	1000 (1000)	0.750		
MABP prior resection*	69 (8)	76 (13)	0.132		
SBP prior resection*	106 (21)	109 (19)	0.570		

Values in parentheses are percentages unless indicated otherwise; ^†^Values are median (iqr); *Values are mean s.d.);

*IVC* inferior vena cava, *PE* pulmonary embolism, *ASA* American Society of Anesthesiologists, *BMI* body mass index, *INR* International normalized ratio, *AST* aspartate aminotransferase, *ALT* alanine aminotransferase, *IVC* inferior vena cava, *PRBC* packed red blood cell, *FFP* fresh frozen plasma, *MABP* mean arterial blood pressure, *SBP* systolic blood pressure, *CVP* central venous pressure, *OR* odds ratio, *CI* confidence interval

Of note, the analysis of general and specific complications following hepatectomy, such as posthepatectomy bile leakage, posthepatectomy liver failure, and posthepatectomy hemorrhage revealed comparable rates between the study groups (Table 4). None of the deaths were attributed to PE.

**Table 4:** Postoperative outcome

	Infrahepatic IVC clamping (*n* = 141)	Control (*n* = 364)	*P* value
Pulmonary embolism	4 (3)	9 (3)	0.762
Cardiac complication	6 (4)	19 (5)	0.820
Deep vein thrombosis	3 (2)	14 (4)	0.420
Acute renal failure	3 (2)	16 (4)	0.302
Abdominal fluid collection	17 (12)	41 (11)	0.876
Postoperative transfusion	16 (13)	35 (9)	0.274
PRBCs †	2 (2)	2 (2–3)	0.908
FFP †	4 (2)	4 (2–7)	0.690
Bile leakage	34 (24)	71 (20)	0.272
Grade B/C	25 (18)	55 (15)	0.378
Posthepatectomy hemorrhage	8 (6)	9 (3)	0.095
Grade B/C	5 (4)	7 (2)	0.397
Posthepatectomy liver failure	14 (10)	31 (8)	0.605
Grade B/C	11 (8)	25 (7)	0.541
Radiological intervention	35 (25)	94 (26)	0.910
Reoperation	19 (14)	39 (11)	0.437
Clavien–Dindo classification			0.932
Grade I	8 (5)	33 (9)	
Grade II	20 (14)	51 (14)	
Grade III	39 (28)	100 (28)	
Grade IV	7 (5)	19 (6)	
Grade V (death)	9 (6)	24 (6)	0.941
Length of postoperative stay (days) †	11 (8–19)	11 (7–20)	0.852

Values in parentheses are percentages unless indicated otherwise;^†^Values are median (iqr); *Values are mean (s.d.)

*IVC* inferior vena cava, *PRBC* packed red blood cell, *FFP* fresh frozen plasma

### Subgroup analysis of patients with major hepatectomy and vascular resections

As major hepatic hepatectomies and vascular resections were more common in the IVC clamping group, we performed a subgroup analysis on the effects of infrahepatic IVC clamping on perioperative outcome of patients who underwent a major hepatectomy with or without vascular resections (Table 5). Again, there was no significant difference in the incidence of postoperative PE between major hepatectomy patients with and without IVC clamping in the vascular resection (3% vs. 2%, *P* = 0.692) and no vascular resection groups (3% vs. 2%, *P* = 0.394). In line with these data, the pooled rate of PE was comparable in the individual studies (OR 1.26, 95%CI 0.45–4.36, *P* = 0.767) as well as in the subgroups with major hepatectomy and vascular resection (OR 1.54, 95%CI 0.42–5.63, *P* = 0.689) or no vascular resection (OR 1.74, 95%CI 0.35–8.56, *P* = 0.498), respectively (Fig. 2 B, C). Intraoperative blood loss was reduced following IVC clamping in both subgroups, though these differences failed to reach statistical significance (*P* = 0.197 and *P* = 0.128). Other intraoperative outcomes were well-balanced between the subgroups. Furthermore, the number of patients with general postoperative complications and specific complications following hepatectomy was well-balanced.

**Table 5:** Subgroup analysis of perioperative outcomes in patients with major hepatic resections

	Vascular resection			No vascular resection		
	Infrahepatic IVC clamping (*n* = 80)	Control (*n* = 166)	*P* value	Infrahepatic IVC clamping (*n* = 93)	Control (*n* = 182)	*P* value
*Intraoperative outcome*						
CVP prior to resection *	5 (3)	5 (3)	0.167	5 (3)	5 (3)	0.197
Extrahepatic resection	16 (17)	24 (13)	0.333	12 (15)	18 (11)	0.314
Bilioenteric anastomosis	23 (25)	47 (25)	0.885	11 (14)	37 (22)	0.125
Pringle maneuver	23 (25)	43 (24)	0.839	20 (12)	38 (23)	0.715
Operating time (min) *	230 (106)	240 (107)	0.443	206 (81)	229 (97)	0.067
Total blood loss (ml) †	1000 (500–1450)	1000 (650–1800)	0.197	900 (500–1400)	1000 (700–1700)	0.128
Mean blood loss	1200 (1100)	1400 (1300)	0.336	1200 (1200)	1400 (1300)	0.515
Intraoperative transfusion	20 (22)	42 (23)	0.879	17 (21)	35 (21)	1.000
PRBCs †	3 (2–4)	2 (2–4)	0.709	3 (2–4)	2 (2–4)	0.330
FFP †	4 (3–5)	4 (2–4)	0.530	4 (3–4)	4 (2–4)	0.372
MABP prior resection *	77 (14)	75 (13)	0.589	76 (13)	76 (13)	0.451
SBP prior resection *	108 (19)	110 (23)	0.561	109 (17)	109 (23)	0.954
*Postoperative outcome*						
Pulmonary embolism	3 (3)	4 (2)	0.692	3 (4)	3 (2)	0.394
Cardiac complication	6 (7)	11 (6)	1.000	6 (8)	9 (5)	0.573
Deep vein thrombosis	3 (3)	8 (4)	0.755	2 (3)	6 (4)	1.000
Acute renal failure	3 (3)	11 (6)	0.395	2 (3)	8 (5)	1.000
Abdominal fluid collection	12 (13)	27 (15)	0.718	9 (11)	22 (13)	0.838
Postoperative transfusion	17 (18)	23 (13)	0.189	12 (15)	20 (12)	0.548
Bile leakage	30 (32)	50 (28)	0.483	27 (34)	45 (27)	0.298
Grade B/C	22 (25)	43 (24)	0.280	20 (25)	38 (23)	0.348
Posthepatectomy hemorrhage	7 (8)	5 (3)	0.078	7 (9)	4 (3)	0.042
Grade B/C	4 (3)	6 (3)	0.106	4 (6)	4 (2)	0.283
Posthepatectomy liver failure	14 (14)	26 (13)	0.858	10 (13)	21 (13)	1.000
Grade B/C	11 (11)	21 (11)	0.751	8 (10)	16 (9)	0.678
Radiological intervention	28 (30)	66 (36)	0.348	22 (28)	52 (31)	0.557
Reoperation	18 (19)	27 (15)	0.389	14 (18)	21 (13)	0.333
Clavien–Dindo classification			0.852			0.713
Grad I	6 (6)	16 (9)		6 (7)	16 (10)	
Grade II	13 (14)	27 (15)		9 (12)	26 (16)	
Grade III	28 (30)	62 (34)		25 (31)	54 (32)	
Grade IV	6 (7)	12 (7)		4 (5)	10 (6)	
Grade V (death)	9 (10)	21 (11)	0.828	6 (8)	17 (10)	0.789
Length of postoperative stay (days) †	15 (9–23)	15 (9–28)	0.501	12 (9–22)	15 (8–25)	0.520

Values in parentheses are percentages unless indicated otherwise; †Values are median (iqr); *Values are mean (s.d.)

*CVP* central venous pressure, *MABP* mean arterial blood pressure, *SBP* systolic blood pressure

## Discussion

Infrahepatic IVC clamping to reduce blood loss was first introduced in 2004 by Otsubo et al. [6] Although this technique was shown to be safe in various cohort studies [23, 24], serious concerns of postoperative PE were raised after the results of a randomized controlled trial were published [8]. Five other randomized trials evaluated the impact of infrahepatic IVC clamping on intraoperative blood loss; however, none but two of these studies outlined postoperative complications in detail [9–12, 19]. Chen et al. included a selective cohort of cirrhotic patients with HCC who underwent mesohepatectomies and detected comparable morbidity rates in infectious complications, pleural effusion, ascites, and hepatic encephalopathy, respectively [19]. Ueno et al. applied a different IVC clamping technique (partial clamping) and reported exclusively on liver-specific complications (e.g., bile leak, pleural-effusion ascites, hyperbilirubinemia) in a majority of patients with primary liver malignancies (79%) and minor hepatectomies (53%) [12]. Therefore, two recent meta-analyses comprising six randomized trials with heterogeneous surgical techniques, perioperative care, and inclusion of selective patient cohorts, failed to give conclusive evidence on this topic [13, 14]. Moreover, Fancellu et al. raised serious concerns in their meta-analysis about a potential type II-error with regard to fewer complications after IVC clamping [14]. The present study addressed this lack of evidence by secondary data analysis of prospective trials including patients who underwent hepatic resections with standardized surgical techniques and perioperative management. The results showed that infrahepatic IVC clamping was neither associated with PE, nor with other postoperative complications. A further subgroup analysis of patients who underwent major hepatic resection with and without vascular resection confirmed these findings.

In previous studies, the onset of PE after liver resection was associated with high BMI, major hepatectomy, liver fibrosis, and previous thromboembolic events [25, 26]. While we detected no independent risk factors for PE, we observed a rather low overall incidence of PE after hepatic resection in our prospectively acquired dataset. While the incidence of PE was 3% in our patients, an incidence of 6% is reported for patients undergoing hepatic resection in the literature [25, 27]. Still, less than half of our patients with PE had peripheral thrombosis suggesting thromboembolic events during liver resection and/or regeneration with accompanying activation of the coagulation cascade [28]. Historically, the risk of venous thromboembolism after hepatectomy was considered to be rare due to decreased postoperative synthesis of clotting factors [29]. However, there is rising evidence of hypercoagulability after hepatobiliary surgery advocating routine perioperative thromboprophylaxis [30]. In the present analysis, all included patients underwent routine thromboprophylaxis. Of note, the rate of clinically relevant posthepatectomy hemorrhage was found to be comparable to other studies [31, 32].

Compared to our previous trial on infrahepatic IVC clamping, we could not detect a significant decrease of total blood loss in patients with infrahepatic IVC clamping [8]. Although two included trials (NCT01858987, NCT00732979) clearly showed a benefit of IVC clamping, in particular, in patients with major hepatectomy, the difference of blood loss was subtle in the other studies resulting in a balanced amount of blood loss between the study groups. However, IVC clamping was performed in four out of five studies irrespective of the CVP prior to resection and on behalf of the surgeons which might have caused heterogenous results. We and other groups previously showed that there is no correlation between CVP and intraoperative blood loss in the range of low CVP values [8, 33]. The Pringle maneuver was only used in 20% of the included patients and limited to cases with significant blood loss. IVC clamping was well-tolerated in the patient cohort with > 84% of the patients having a clamping-time > 5 min. Unfortunately, we could not assess blood loss during hepatic transection in the patient cohort, which might have been the more suitable outcome parameter addressing this question. Further, the study and control groups had significant discrepancies regarding the extent of hepatectomy and vascular resections. Although we performed subgroup analyses to adjust the study and control groups, the effect of IVC clamping could have been reduced due to other non-balanced unknown factors.

There are some limitations to the present study. First, this was a secondary data analysis of available data from prospective trials of our study group prohibiting definitive conclusions. In fact, only a few trials addressed the impact of IVC clamping in liver surgery so far and, in particular, there is lacking data in patients with secondary liver malignancies. Therefore, large-scale multi-institutional studies are needed to provide conclusive results. Second, we assessed the impact of IVC clamping on a selected patient cohort without severe comorbidities, advanced liver cirrhosis, or known coagulopathies. This might have caused some selection bias. Third, all patients underwent conventional hepatic resection. As minimally invasive surgery is an emerging field in liver surgery, the results of the present study might not be transferable to laparoscopic resections because pneumoperitoneum and patient positioning may result in unreliable CVP values [34, 35]. Although a recent randomized trial demonstrated that lowering of CVP values in laparoscopic hepatectomy was associated with lower blood loss, IVC clamping was omitted in this trial and the safety and impact in minimally invasive surgery remains unclear [36]. Fourth, we did not include studies with the restricted use of, e.g., anterior-approach hepatectomy for large primary liver malignancies. Therefore, our findings should be interpreted with caution in other patient cohorts.

## Conclusion

We found no adverse impact of IVC clamping during conventional hepatic resection. In particular, there was no association with PE after elective hepatic resection in the present study cohort. Infrahepatic IVC clamping may therefore be applied as a safe technique to reduce CVP in patients undergoing elective hepatic resection.
